# A new mammal from the Lower Cretaceous Jehol Biota and implications for eutherian evolution

**DOI:** 10.1098/rstb.2021.0042

**Published:** 2022-03-28

**Authors:** Hai-Bing Wang, Simone Hoffmann, Dian-Can Wang, Yuan-Qing Wang

**Affiliations:** ^1^ Key Laboratory of Vertebrate Evolution and Human Origins of Chinese Academy of Sciences, Institute of Vertebrate Paleontology and Paleoanthropology, Beijing, People's Republic of China; ^2^ Centre for Excellence in Life and Paleoenvironment, Chinese Academy of Sciences, Beijing, People's Republic of China; ^3^ Key State Key Laboratory of Palaeobiology and Stratigraphy, Nanjing Institute of Geology and Palaeontology, Chinese Academy of Sciences, Nanjing, People's Republic of China; ^4^ Department of Anatomy, College of Osteopathic Medicine, New York Institute of Technology, Old Westbury, NY, USA; ^5^ Department of Oral and Maxillofacial Surgery, Peking University School of Stomatology, 22 South Zhongguancun Avenue, Beijing, People's Republic of China; ^6^ College of Earth and Planetary Sciences, University of Chinese Academy of Sciences, Beijing, People's Republic of China

**Keywords:** Early Cretaceous, Jehol Biota, Eutheria, Meckelian cartilage, inner ear, tooth replacement

## Abstract

Here we report on a new Early Cretaceous eutherian represented by a partial skeleton from the Jiufotang Formation at Sihedang site, Lingyuan City, Liaoning Province that fills a crucial gap between the earliest eutherians from the Yixian Formation and later Cretaceous eutherians. The new specimen reveals, to our knowledge for the first time in eutherians, that the Meckelian cartilage was ossified but reduced in size, confirming a complete detachment of the middle ear from the lower jaw. Seven hyoid elements, including paired stylohyals, epihyals and thyrohyals and the single basihyal are preserved. For the inner ear the ossified primary lamina, base of the secondary lamina, ossified cochlear ganglion and secondary crus commune are present and the cochlear canal is coiled through 360°. In addition, plesiomorphic features of the dentition include weak conules, lack of pre- and post-cingula and less expanded protocones on the upper molars and height differential between the trigonid and talonid, a large protoconid and a small paraconid on the lower molars. The new taxon displays an alternating pattern of tooth replacement with P3 being the last upper premolar to erupt similar to the basal eutherian *Juramaia*. Parsimony analysis places the new taxon with *Montanalestes*, *Sinodelphys* and *Ambolestes* as a sister group to other eutherians.

This article is part of the theme issue ‘The impact of Chinese palaeontology on evolutionary research’.

## 1. Introduction

**Figure 1.**
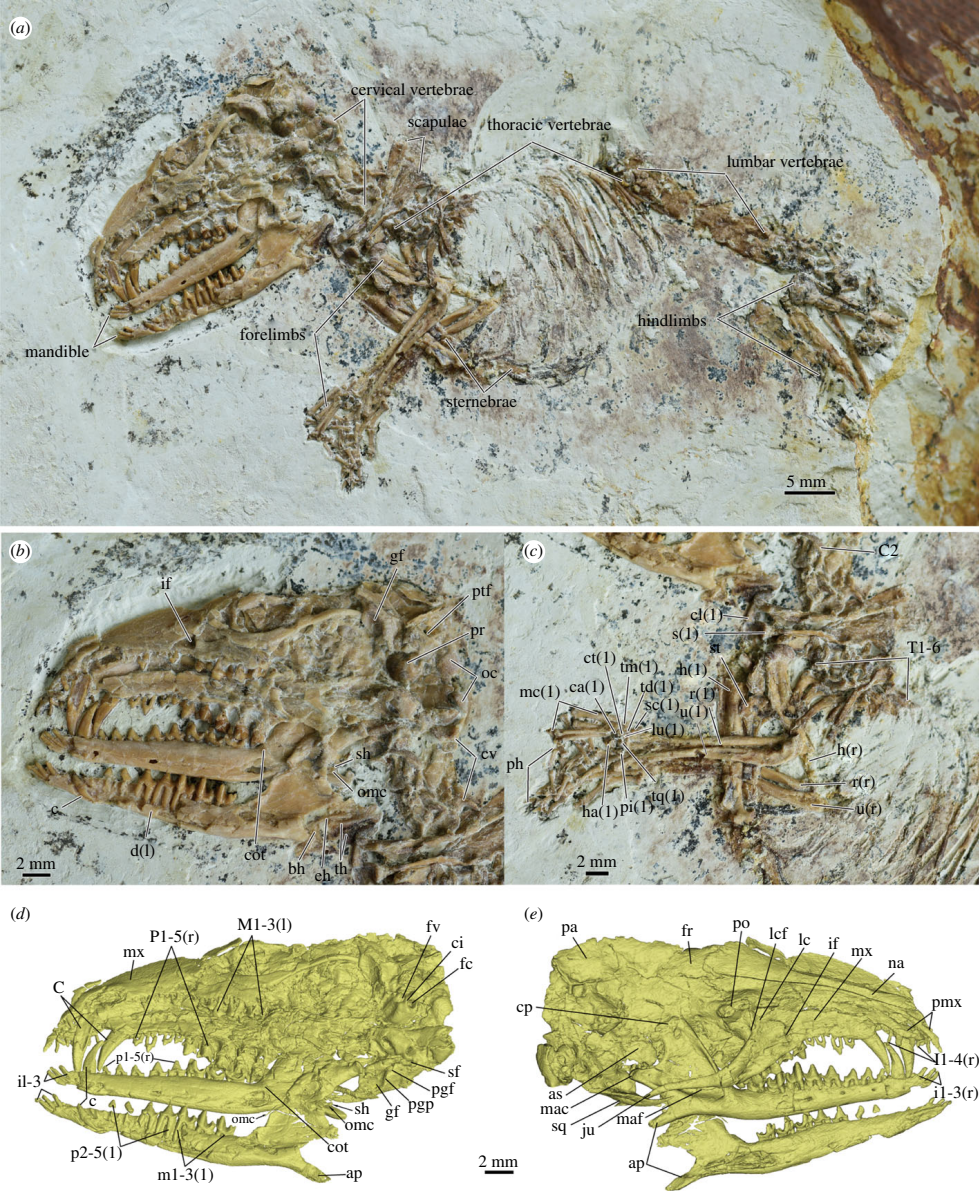
Holotype specimen of *Cokotherium jiufotangensis* (IVPP V23387). (*a*) Skeleton of *Cokotherium jiufotangensis*; (*b*) skull in ventrolateral view; (*c*) forelimb mainly in lateral view; (*d*) virtual reconstruction of the skull in dorsolateral view; (*e*) virtual reconstruction of the skull in ventrolateral view. Right side indicated by (r), left side indicated by (l). (*d*) and (*e*) at same scale. ap, angular process; as, alisphenoid; bh, basihyal; c, lower canine; C, upper canine; C2, axis (cervical vertebra 2); ca, capitate; ci, crista interfenestralis; cl, clavicle; cot, coronoid tubercle; cp, coronoid process; ct, centrale; cv, cervical vertebrae; d, dentary; eh, epihyal; fc, fenestra cochleae; fr, frontal; fv, fenestra vestibuli; gf, glenoid fossa; h, humerus; ha, hamate; if, infraorbital foramen; ju, jugual; lc, lacrimal; lcf, lacrimal foramen; lu, lunate; mac, mandibular condyle; maf, masseteric fossa; max, maxilla; mc, metacarpals; na, nasal; oc, occipital condyle; omc, ossified Meckelian cartilage; pa, parietal; pgf, postglenoid fossa; pgp, postglenoid process; ph, phalanges; pi, pisiform; pmx, premaxilla; po, postorbital process; pr, promontorium; ptf, posttemporal foramen; r, radius; s, scapular; sc, scaphoid; sh, stylohyal; sf, stapedius fossa; sq, squamosal; st, sternum; T, thoracic vertebrae; td, trapezoid; th, thyrohyal; tm, trapezium; tq, triquetrum; u, ulnar.

Placentals are the most specious and diverse of the three living mammalian clades [1] and their evolutionary history can be traced back more than 120 Myr to the Mesozoic era [2]. Most molecular estimates, while covering a broad temporal range, place the divergence of eutherians (placentals and their closest relatives) and metatherian (marsupials and their closest relatives) into the Jurassic period [3–5]. Such an early separation of the two therian clades is supported by a single fossil, *Juramaia*, currently considered the earliest eutherian and the only one known prior to the Cretaceous ([6], but see [7] for a critical evaluation of the geological age of *Juramaia*). Aside from the possible Late Jurassic *Juramaia*, the fossil record of early eutherians is sparse and only a handful of well-preserved specimens are known from the Early Cretaceous, including *Eomaia* [8], *Acristatherium* [9], *Amboleste*s [10], *Sasayamamylos* [11], *Sinodelphys* [10,12] and *Prokennalestes* [13]. The early evolutionary history of metatherians is even less well documented [10,14,15]. Further complicating the understanding of the divergence of metatherians and eutherians is the fact that characteristics historically used to define each clade (e.g. molar count, size of carpals) have recently come under question with the placement of the previously earliest metatherian, *Sinodelphys*, within Eutheria [10]. Gaining a better understanding of the evolution of early eutherians relies on fossil discoveries in the Late Jurassic and Early Cretaceous that are based on associated cranial, mandibular and postcranial material. Here we report a new basal eutherian consisting of a partial skeleton from the Lower Cretaceous Jiufotang Formation, Jehol Biota. The cranium is almost complete, including the basicranium and ear region (figure 1). This specimen provides polarity for a series of characters important in defining the metatherian/eutherian lineages. This includes characters regarding dental and inner ear morphology. Aided by micro-computed tomography (CT), we provide, to our knowledge for the first time, a virtual reconstruction of the inner ear of an Early Cretaceous eutherian. Several changes in inner ear morphology (e.g. presence of ossified primary and secondary laminae, ossified ganglion canal, absence of a lagena macula) have been reconstructed for Cladotheria based on the presences of these features in dryolestoids and stem therians [16–19]. However, direct evidence from Early Cretaceous therians is still limited. Based on the new fossil material, we are able to document and evaluate inner ear morphology in a basal eutherian.

## 2. Methods

The holotype specimen, IVPP V23387, is a partial skeleton. The specimen was prepared from the left side which exposed the skull in ventrolateral view and much of the postcranial skeleton in a roughly left lateral view. The specimen was scanned using a Zeiss Xradia Versa 520 X-ray CT at the Institute of Process Engineering, Chinese Academy of Sciences, Beijing (140 kV, 71 µA, voxel size = 30.289 µm). Digital preparation was performed in VGStudio v. 3.0 and Amira 2020.1. The new taxon was scored into the character taxon matrix of Bi *et al*. [10] (see the electronic supplementary material). Maximum-parsimony analysis was performed in Tree analysis using New Technology (TNT) 1.5 [20], using the New Technology search algorithm with sectorial search, ratchet (200 iterations) [21], tree drift (100 cycles) and tree fusing (100 rounds) [22]. Trees were rooted along the branch leading to *Thrinaxodon*. All characters were treated as equally weighted and unordered and a strict consensus (Nelson consensus) tree was calculated in TNT. In addition, an undated Bayesian analysis was performed in MrBayes v. 3.2 [23]. The Bayesian analysis ran for 50 million Markov chain Monte Carlo generations with the first 25% discarded as ‘burn-in’, using the Mkv model for discrete morphological data and a gamma parameter for rate variation. Post-burn-in trees are summarized in 50% majority-rule consensus trees (halfcompat). Bremer support values and posterior probabilities are used to gauge node support in the tree topologies of the parsimony and Bayesian analyses, respectively.

## 3. Results

**Systematic palaeontology**

**Class** Mammalia Linnaeus, 1758

**Infraclass** Eutheria *sensu* Huxley, 1880

**Order** incertae sedis

**Family** incertae sedis

*Cokotherium* gen. nov.

*Cokotherium jiufotangensis* sp. nov.

**Etymology**. Coko, in reference to the given name of the late palaeontologist Chuan-Kui Li (C. K. Li) for his contribution in understanding the evolution of early mammals; the specific name denotes that the Jiufotang Formation, the upper part of the lacustrine deposits producing the Jehol Biota, where the holotype and the first reported specimen was collected.

**Holotype**. A partial skeleton with complete skull, forelimbs and part of the trunk and hindlimbs preserved on one slab (IVPP V23387) (figure 1).

**Locality and horizon**. The holotype was discovered in the Lower Cretaceous Jiufotang Formation at the Sihedang site, Lingyuan City, Liaoning Province, dated around 120 Ma [24].

**Differential diagnosis**. Dental formula: I4-C1-P5-M3/i3-c1-p5-m3 (I: incisor; C: canine; P, premolar: M, molar; upper and lower cases denote upper and lower teeth, respectively), which is typical for Early Cretaceous eutherian postcanine dentition (five premolars and three molars). Vestigial ossified Meckelian cartilage; elongate and posteriorly extended angular process. Molars tribosphenic with weak development of conules, reduced stylar shelves on the upper molars; with wide talonids bearing distinct entoconid and hypoconulid on the lower molars. Derived characteristics include semimolarized, three-rooted P5 and lower count of upper/lower incisors (compared to contemporary eutherians). *Cokotherium* differs from basal eutherians in having an elongate and posteriorly directed angular process and, with the exception of *Sinodelphys*, a small trapezium. Among Jurassic-Early Cretaceous eutherians, *Cokotherium* has the same dental formula as *Acristatherium*, but differs in having single-rooted canine, semimolarized P5, narrow stylar shelves, longer and wider talonid and reduction of the septomaxilla. Differs from *Ambolestes* in having single-rooted upper canine, eight postcanine teeth, semimolarized P5, transversely wide upper molars and seven hyoid bones. Differs from *Sinodelphys* in having fewer incisors and tightly implanted premolars. Differs from *Montanalestes* in having paraconid lower than metaconid. Differs from *Juramaia* in having four upper and three lower incisors, single-rooted canine, wide protoconal region and Meckelian sulcus. Differs from *Eomaia* in having an elongate angular process, four upper and three lower incisors, non-trenchant P5, longer trigonid in lower molars. Differs from *Murtoilestes* in having less-developed conules in upper molars and larger M1 relative to M2. Differs from *Sasayamamylos* in having five lower premolars, Meckelian sulcus, mandibular condyle more posteriorly situated.

**Figure 2.**
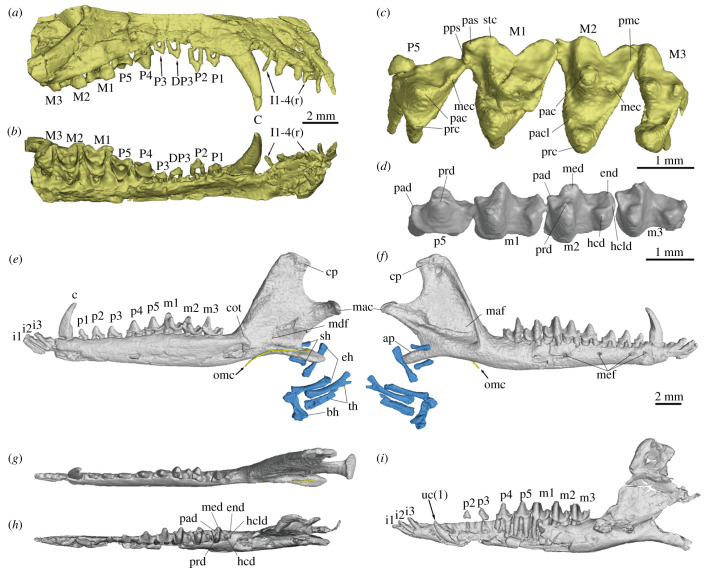
Jaws and dentition of *Cokotherium jiufotangensis* (IVPP V23387). (*a*) Right upper jaw in lateral view; (*b*) right upper jaw in occlusal view; (*c*) left P5-M3 in occlusal view; (*d*) left p5-m3 in occlusal view; (*e*) right mandible with the ossified Meckelian cartilage (yellow) and hyoid bones (blue) in medial view; (*f*) right mandible with the ossified Meckelian cartilage (yellow) and hyoid bones (blue) in lateral view; (*g*) right mandible in dorsal view; (*h*) left mandible in dorsal view; (*i*) left mandible in lateral view showing unerupted canine. Right side indicated by (r), left side indicated by (l). (*e*)-(*i*) at same scale. ap, angular process; bh, basihyal; c, lower canine; C, upper canine; cot, coronoid tubercle; cp, coronoid process; DP, deciduous upper premolar; eh, epihyal; end, entoconid; hcd, hypoconid; hcld, hypoconulid; i, lower incisor; I, upper incisor; m, lower molar; M, upper molar; mac, mandibular condyle; maf, masseteric fossa; mdf, mandibular foramen; mec, metacone; med, metaconid; mef, mental foramen; omc, ossified Meckelian cartilage; p, lower premolar; P, upper premolar; pac, paracone; pacl, paraconule; pad, paraconid; pas, parastyle; pmc, post-metacrista cusp; pps, preparastyle; prc, protocone; prd, protoconid; sh, stylohyal; stc, stylocone; th, thyrohyal; uc, unerupted lower left canine.

**Description**. The skull is exposed in ventrolateral view, but the dorsal and right lateral aspects of the cranium are visible in the CT images (figures 1 and 2a). The septomaxilla is absent. The premaxilla is slender and extends posteriorly to the level of the upper canines. Dorsally its facial process contacts the nasal along its whole length. The nasal is slender and its length is just over half of the cranial length. The nasal gradually expands posteriorly. The nasofrontal suture extends posterior to the anterior orbital rim and is concave anteriorly. A single, large lacrimal foramen is present within the orbit just medially to the orbital margin (figure 1*e*). The maxilla has no contact with the frontal. A sizeable maxillary foramen is present, but it is unclear which bones border the foramen. The maxillary foramen leads to a large and single infraorbital foramen within the maxilla (figure 1*b,e*). The zygomatic arch is delicate with contributions from the jugal anteriorly and the squamosal posteriorly. The zygomatic process of the maxilla is vestigial. The posterior edge of the anterior zygomatic root is aligned with the ultimate molar. The lateral wall of the braincase and most of the orbit are badly damaged and the many cracks obscure the morphology. The frontal is slightly shorter than the parietal in anteroposterior direction. The frontoparietal suture is anteriorly concave. A small postorbital process is visible near the right nasofrontal suture, on the left side it is obscured by a crack. The sagittal crest is gently elevated and the posttemporal foramen is large (figure 1*b*). In ventral view, the palatine is badly damaged and partly covered by the left maxilla and jugal (figure 1*b,d*). The palatine extends anteriorly up to the level of P5. The posterior aspect of the palatine is preserved; the postpalatine torus is present and the posterior nasal spine is weak. The pterygoid is shallow and has a hamulus.

**Figure 3.**
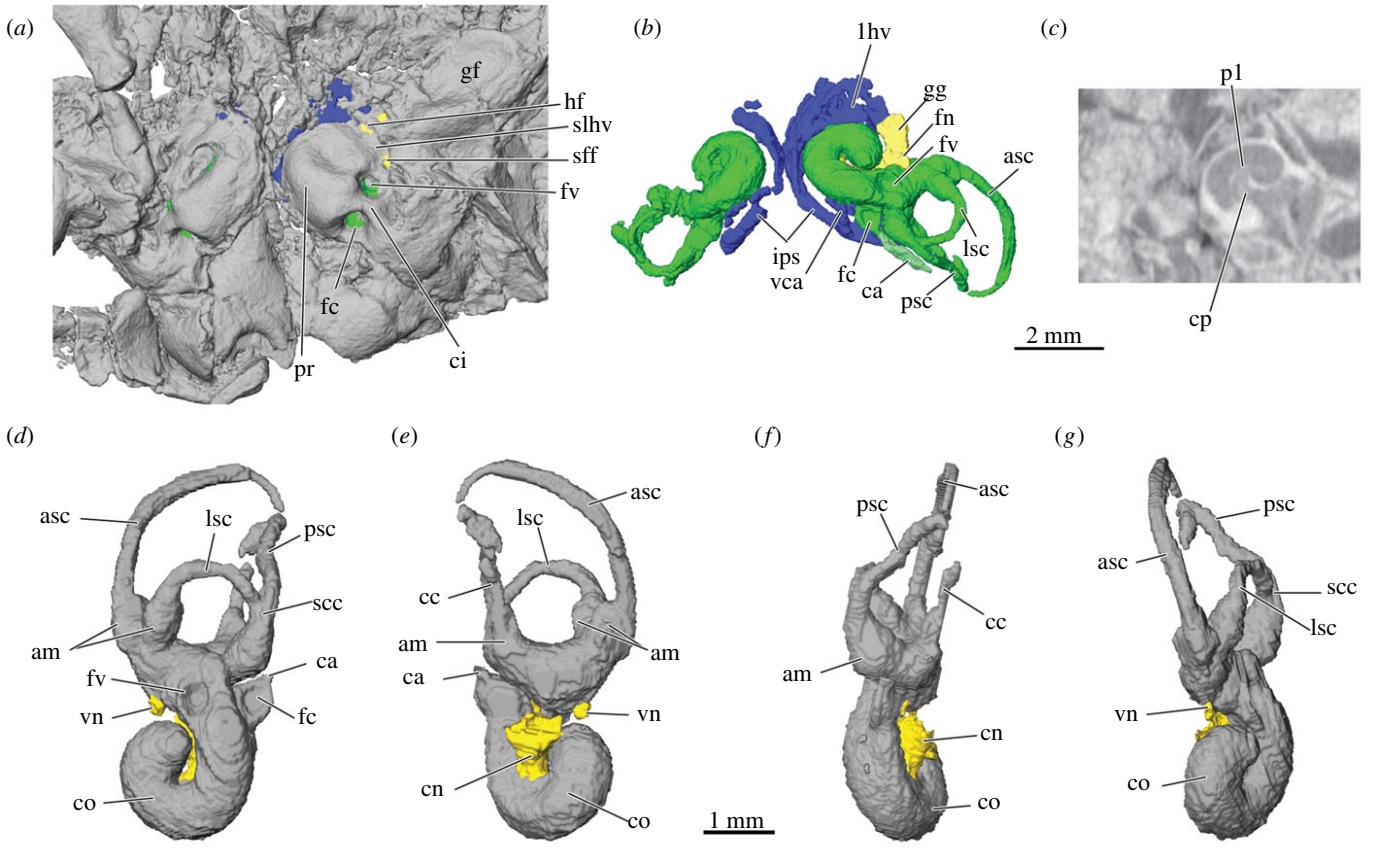
Inner ear of *Cokotherium jiufotangensis* (IVPP V23387). (*a*) Position of inner ear (green), veins (blue), and nerves (yellow) in ventral view of cranium; (*b*) endocast of right and left inner ear (green), veins (blue), and nerves (yellow) in same ventral view; (*c*) cross section through left inner ear showing internal structures of cochlear canal; (*a-c*) at same scale. Endocast of inner ear (grey), cochlear and vestibular nerves (yellow) in (*d*) ventral, (*e*) dorsal, (*f*) medial and (*g*) lateral views, anterior is down, all at same scale. am, ampulla; asc, anterior semicircular canal; ca, cochlear aqueduct; cc, crus commune; ci, crista interfenestralis; cn, cochlear nerve; co, cochlear canal; cp, cribriform plate; crp, crista parotica; fc, fenestra cochleae; fn, facial nerve; fv, fenestra vestibuli; gf, glenoid fossa; gg, geniculate ganglion; hf, hiatus Fallopii; ips, inferior petrosal sinus; jf, jugular fossa; lhv, lateral head vein; lsc, lateral semicircular canal; pl, primary osseous lamina; pr, promontorium; psc, posterior semicircular canal; scc, secondary crus commune; sff, secondary facial foramen; slhv, sulcus for lateral head vein; vca, vein of cochlear aqueduct; vn, vestibular nerve.

The basicranial region is slightly compressed transversely, but the pars cochlearis is generally well-preserved (figure 3). In ventral view, the promontorium is bulbous. The ventral surface of the promontorium does not bare any vascular grooves for the internal carotid. A shallow groove appears to be present medial to the promontorium indicating an extrabullar course of the internal carotid artery. A slender sulcus passing from the fenestra vestibuli laterally along the promontorium is here identified as the stapedial sulcus. The stapedial artery probably passed from the internal carotid artery laterally through the stapes and then with the lateral head vein anteriorly along the lateral aspect of the promontorium similar to the reconstructions for *Zalambdalestes* [25]. Lateral to the promontorium and posterior to the hiatus Fallopii is the fossa for the tensor tympani muscle. The fenestra vestibuli and the fenestra cochleae are well defined in ventral view. The fenestra cochleae is nearly circular whereas the fenestra vestibuli is oval. The perilymphatic duct is separate and enclosed in bone forming an aqueductus cochleae. Thus, a true fenestra cochleae is present in *Cokotherium*. The aqueductus cochleae passes posteriorly and opens along the lateral aspect of the jugular foramen. The crista interfenestralis, separating the fenestra cochleae and the fenestra vestibuli, is narrow in ventral view and connects to a small and gently rounded paraoccipital process posteriorly. A deep trough that is bordered medially by the crista interfenestralis and the promontorium and laterally by the crista parotica extends anteriorly from the paroccipital process. At the posterior aspect of the trough and lateral to the crista interfenestralis is a subtle and round stapedius fossa. Within the sulcus lie, from posterior to anterior, the secondary facial foramen, hiatus Fallopii and the opening for the prootic sinus. The trough probably carried the stapedial artery and the lateral head vein. Openings for the ramus superior and ramus inferior of the stapedial artery could not be clearly identified because of crushing. Based on micro-CT images, the course of the facial nerve could be traced from the internal acoustic meatus to the cavum supracochleare, which housed the geniculate ganglion. The cavum suprachochleare has a bony roof with a secondary facial foramen as the cranial exit of the facial nerve. Anteriorly, the cavum suprachochleare connects to the hiatus Fallopii, the opening for the greater petrosal nerve. A piriform fenestra could not be confidently identified. The glenoid fossa is concave, mediolaterally wider than anteroposteriorly long, and positioned partially on the braincase. The postglenoid process is small and posterior to it is a postglenoid foramen (figure 1*d*). On the left side, a single hypoglossal foramen can be identified anterior to the occipital condyle.

The bony labyrinth is fairly well preserved and was virtually reconstructed based on micro-CT images. The cochlear canal of *Cokotherium* is coiled to nearly 360°, the perilymphatic duct is enclosed in its own bony channel and opens separately from the fenestra cochleae (figure 3). *Cokotherium* possesses a primary osseous lamina, base of the secondary osseous lamina and bony cribriform plate. The semicircular canals are large and slender. Although partly distorted, it is evident that the posterior and anterior semicircular canals have a much larger radius than the lateral semicircular canal. A crus commune and secondary crus commune are present. An extensive network of veins surrounds the cochlear canal and drains into the inferior petrosal sinus and prootic vein (figure 3*b*). Similar to extant therians the vein of the cochlear aqueduct drains the cochlea along the base of the secondary lamina and connects to the inferior petrosal sinus.

The middle ear bones could not be identified with confidence. A ring-like element situated near the right mandibular condyle might represent part of the ectotympanic. The fragmentary nature of the element makes the identification tentative at best. In addition, part of the malleus might be preserved close to the mandibular condyle of the right mandible with a ring-like outline in scan images. By contrast, most elements of the hyoid apparatus can be identified in *Cokotherium*, including the paired stylohyals, epihyals and thyrohyals and the single basihyal (figures 1 and 2). The thyrohyal is characteristically expanded at its distal end. The stylohyal and epihyal are more rod-like than the thyrohyal, with the stylohyal being shorter than the epihyal.

The right mandible is nearly complete, only sustaining some damage at the anterior tip (figures 2*e–i*). The left mandible is slightly less well preserved, with some damage on the lateral aspect of the horizontal ramus, coronoid process, mandibular condyle and medial aspect of the angular process (figure 2*h,i*). The mandible is slender and has a slightly concave ventral edge anterior to the angular process. The bone surrounding the alveoli extends further dorsally on the lateral aspect of the mandible than on the lingual aspect. At least four mental foramina are present on the right mandible, whereas the lateral aspect of the left horizontal ramus is too damaged to determine the number of foramina (figure 2*f,i*). The mandibular symphysis extends posteriorly up to the level of the lower canine. Medially at the junction of the horizontal and ascending rami is a process, here interpreted as the coronoid tubercle (figures 1*b,d* and 2*e*), presumably the vestigial of the coronoid bone [26,27]. The angular process is low, elongate and posteroventrally directed (figure 2*e–f*). The root of the angular process is anteroposteriorly situated at the level of the coronoid process. The mandibular condyle is transversely wide and well defined with a distinct neck (figure 2*d–f*). The masseteric fossa is broad and deep. The coronoid process is broad and tall with its height being nearly half the length of the mandibular ramus. A slender bony element is preserved on the medial aspect of the right angular process, which we interpret to be the right ossified Meckelian cartilage. The anterior part of the ossified Meckelian cartilage appears displaced ventrally as it extends past the mandible. In life, it probably passed along the medial aspect of the mandible onto the horizontal ramus inferior to the coronoid tubercle. The posterior end of the ossified Meckelian cartilage is rounded, probably indicating the absence of any articulation with the middle ear bones. Damage to the medial aspect of the mandible precludes any definite identification of a Meckelian sulcus. Given the small size of the ossified Meckelian cartilage it is likely that the Meckelian sulcus in *Cokotherium* was likewise small, probably similar in size to that of *Ambolestes* and *Prokennalestes*.

Four relatively small upper incisors (I) are present in the premaxilla that are sub-equal in size (figure 2*a*). Large diastemata separate the upper incisors. I3 and I4 are slightly more vertically implanted than I1 and I2. The upper canine is the largest tooth and nearly vertically implanted. Posterior to the upper canine is a short diastema. Nine upper postcanine loci are present, six for the upper premolar (P) series and three for the upper molars (M). The third position among the upper premolar series is the deciduous P3, as in *Juramaia*. Thus, the upper premolar series consists of P1, P2, DP3, P3, P4 and P5 posteriorly. All upper premolars are double-rooted except the P5, which has three roots. The P1 is smaller and slightly more procumbent than the P2, which is the largest of the anterior upper premolars (P1-3). A short diastema is present between P2 and DP3. P3 is the smallest tooth among the postcanines and erupted posterodorsal to the DP3. The penultimate upper premolar, P4, is tall and pointed (trenchant) without either a protocone or protoconal swelling. The P5 has three roots and its tooth crown is transversely expanded, but still narrower than that of M1 (figure 2*c*). A protocone is present on the lingual side of the P5. Laterally, the stylar shelf of P5 is developed and the metastylar lobe is larger than the parastylar lobe in the P5. The three upper molars are labio-lingually wider than antero-posteriorly long and decrease in size posteriorly, with M3 being distinctly smaller than M1 and M2. The outline of the tooth crown of the M1-2 in occlusal view is nearly triangular. The stylar shelf is relatively narrow transversely with the labio-lingual width of the stylar shelf less than half of the total upper molar width. The stylar shelf is most distinct in the M2. The preparastyle and postmetacrista cusp are present in the M1-2. The preparacrista is distinct, connecting the paracone and the stylocone in M1-2. The paracone is slightly larger than the metacone in the upper molars. The paraconule and metaconule are weak and the former is more distinct than the latter in the M2. The protocone is low and lacks an antero-posterior expansion and the hypocone is absent in all three upper molars. The metastylar lobe is absent on the M3.

The three lower incisors are procumbent, tightly implanted and sub-equal in size (figure 2*e,f,i*). The right lower canine is enlarged, single-rooted and vertically implanted. On the left side, the lower canine is not fully erupted (figure 2*i*), and it is interpreted as a deciduous lower canine based on its smaller size (compared to the right lower canine) (see Discussion). There are eight lower postcanines with five lower premolars (p) and three lower molars (m) that are all fully erupted. All lower premolars are double-rooted and increase in size posteriorly. The ultimate lower premolar (p5) is more similar in morphology to p3-p4 than to the lower molars and is, therefore, not semimolariform (figure 2*d*). The heel becomes higher and wider from p3 to p5 posteriorly but never develops a basin. The three lower molars are similar in size (figure 2*d*). The protoconid is taller than the paraconid and metaconid in the lower molars. The metaconid is taller and more robust than the paraconid. The anterolabial cingulid is distinct in the lower molars. The talonid of the lower molars is well-developed. It is similar in antero-posterior length to the trigonid and nearly as medio-laterally wide. The hypoconulid is positioned transversely between the hypoconid and the entoconid. The entoconid, the only cusp on the lingual aspect of the talonid, is more distinct on m1 and m2 than on m3.

The cervical vertebrae, anterior thoracic vertebrae (T1-T6), most lumbar vertebrae, posterior ribs, partial sternum, pectoral girdle and forelimbs as well as partial hind limbs are preserved (figure 1*a,c*). The scapula has a prominent coracoid process that contacts the partially preserved distal end of the clavicle (figure 1*c*). The prominent greater tubercle of the humerus is even to the head of the humerus. Distally the greater tubercle extends into a well-developed deltoid tuberosity that ends roughly at the mid-point of the humeral shaft. The radial notch of the ulnar is triangular in anterior view and the styloid process of the radius is weak. The scaphoid is slightly distally displaced, compressed dorsoventrally and wider than the small lunate. The triquetrum is large and has a triangular outline in anterior view. The pisiform is robust with a rounded distal process. In the distal row, the trapezium is small compared to the other carpals and the trapezoid is compressed laterally. The centrale is exposed in dorsal view and overlaps the capitate. The hamate is the largest carpal bone at the lateral side of the wrist.

## 4. Discussion

### (a) Ossified Meckelian cartilage and middle ear evolution

The Meckelian sulcus is a common feature for most Jurassic-Early Cretaceous mammaliaforms with the exception of *Juramaia* [6], *Sasayamamylos* [11], *Lactodens* [27], euharamiyidans [28], multituberculates [29] and gondwanatherians [30]. Presence of a Meckelian sulcus has been reported in early eutherians, including *Eomaia, Prokennalestes, Hovurlestes* and *Ambolestes* [8,10,13,31], implying that early eutherians retained a Meckelian cartilage similar to the contemporary eutriconodontans and zhangheotheriids. *Cokotherium* provides, to our knowledge for the first time, direct evidence of an ossified Meckelian cartilage in Early Cretaceous eutherians. Among the earliest eutherians, the Meckelian sulcus is reduced in size extending from below the m3 to the mandibular foramen in *Eomaia* [8] and typically in *Prokennalestes* [13], and being confined to below the mandibular foramen in *Ambolestes* [10]. Some variation in size is noted for *Prokennalestes* with the Meckelian sulcus even being absent in at least one specimen [13]. In *Cokotherium*, the ossified Meckelian cartilage is slender and short, extending from below the anterior border of the ascending ramus of the mandible to the dorsal surface of the angular process. A corresponding Meckelian sulcus could not be identified with confidence in IVPP V23387. The size of the ossified cartilage does however fit with the size of the Meckelian sulcus in those early eutherians and is distinctly different from the rod-like and relatively robust ossified Meckelian cartilage of eutriconodontans and zhangheotheriids.

The reduced size of the ossified Meckelian cartilage in *Cokotherium* suggests that the middle ear bones are detached from the cartilage as well as the mandible. The morphology in *Cokotherium* represents an intermediate step between the Meckelian-attached middle ear of eutriconodontans [28,32] in which the middle ear is attached to the mandible by the Meckelian cartilage and the detached middle ear of therians in which Meckelian cartilage is absorbed and the middle ear bones are disconnected from the mandible [28]. The condition of *Cokotherium* is perhaps most reminiscent of the zhangheotheriid *Origolestes* which has an ossified Meckelian cartilage that is separated from the middle ear by a small gap [33] (but see [28] for an alternative interpretation). In comparison to *Origolestes* the Meckelian cartilage in *Cokotherium* is even more reduced in length and diameter and the gap between the middle ear and cartilage was probably even larger. The presence of a reduced ossified Meckelian cartilage in the early eutherian *Cokotherium* illustrates a gradual reduction of the ossified Meckelian cartilage in Mesozoic mammals [34–38].

### (b) Inner ear evolution

*Cokotherium* presents, to our knowledge, the first three-dimensional reconstruction of the inner ear for an Early Cretaceous eutherian that is based on high-resolution CT imaging. The petrosal morphology has been described for the Early Cretaceous *Prokennalestes* (including inferences about the osseous labyrinth) [39] but without CT capability to study the internal aspect of the petrosal. *Cokotherium* thus bridges a gap between inner ear morphology of stem therians (e.g. dryolestoids, *Vincelestes*, Höover petrosals) [17–19,40] and Late Cretaceous eutherians including *Kulbeckia*, *Zalambdalestes*, *Ukhaatherium*, *Uchkudukodon,* zhelestids and eutherians from Bug Creek Anthills [41–45]. Similar to most Cretaceous eutherians the cochlear canal of *Cokotherium* completes a single coil (360°). This fairly consistent degree of coiling is only exceeded by some zhelestids and *Kulbeckia*. *Cokotherium* confirms the presence of several features thought to be plesiomorphic for eutherians, such as a secondary crus commune, base of a secondary osseous lamina, primary osseous lamina and a bony cribriform plate. By contrast to *Prokennalestes* and other basal cladotherians, *Cokotherium* has an extrabullar course of the internal carotid artery similar to that of the Late Cretaceous *Ukhaatherium*, *Asioryctes*, *Kennalestes* and *Zalambdalestes* [10]. *Cokotherium* retains the plesiomorphic condition of a large inferior petrosal sinus that wraps around the cochlear canal similar to more basal mammals [19,46,47]. An intrapetrosal inferior petrosal sinus has also been reconstructed for *Prokennalestes* but a comparable structure is not known for extant therians [39]. In addition, *Cokotherium* probably retained a prootic sinus that connected the inferior petrosal sinus to the lateral head vein. By contrast to these plesiomorphic features, *Cokotherium* is derived and similar to extant therians and stem therians described by Harper & Rougier [19] in that the vein of the cochlear aqueduct drains the cochlea by passing through the base of the secondary lamina to the inferior petrosal sinus.

### (c) Dentition

*Cokotherium* differs from the stem therian *Kielantherium* in having three instead of four lower molars and from *Aegialodon* in having a wider talonid and a paraconid that is smaller than the metaconid. It is distinct from metatherians in having three upper and lower molars, size differentiation between paraconid and metaconid, and in lacking the inflected angular process, keel-like paraconid on the lower molars (typically seen in deltatheroidans) and twinning of hypoconulid and entoconid on the lower molars (typically seen in *Alphadon*) [2]. *Cokotherium* exhibits a combination of dental features that is distinct from other Jurassic and Cretaceous eutherians. *Cokotherium* retains several features thought to be plesiomorphic for eutherians including a generalized conical morphology of the incisors, presence of five upper and five lower premolars, weak conules on the upper molars, lack of pre- and post-cingula on the upper molars, a less expanded protocone on the upper molars that is still distinctly larger than the metacone, distinct height differential between the trigonid and talonid, protoconid on lower molars larger than the paraconid and metaconid and a paraconid that is smaller than the metaconid. By contrast to those basal features *Cokotherium* is derived in having a reduced number of upper and lower incisors (four upper incisors and three lower incisors) in comparison to *Eomaia* and *Juramaia* (five upper and four lower incisors) and *Sinodelphys* (four upper and lower incisors each). Among early eutherians, the premolars are closely packed in *Juramaia* but further spaced out, similar to those of *Cokotherium*, in *Eomaia*, *Acristatherium* and *Sasayamamylos*. Interestingly, the upper canine is single-rooted in *Cokotherium* similar to that of most early metatherians but in contrast to the double-rooted canine in *Juramaia*, *Acristatherium* and *Ambolestes* [6,9,10]. *Cokotherium* also possesses a single-rooted lower canine, but this feature is more widely distributed in early eutherians with the exception of *Prokennalestes* and *Sasayamamylos* [11].

*Cokotherium* displays an intriguing pattern of molarization of the premolars. The ultimate upper premolar is molarized with three roots, a lingual protoconal swelling and a metaconal swelling, whereas the ultimate lower premolar is non-molarized. This is similar to the condition in *Prokennalestes*, but differs from that in most other eutherians. Molarization of the ultimate premolars is generally considered a derived feature present in most Late Cretaceous eutherians but lacking in early Cretaceous eutherians. The incipient metacone and protocone in *Cokotherium* and *Prokennalestes* is also present in some Late Cretaceous eutherians, such as zhelestids, asioryctitherians, cimolestids and zalambdalestids [25,48–50]. However, the protocone in the ultimate upper premolar is confined close to the principal cusp (paracone) in *Cokotherium* instead of distant from the paracone as shown in Late Cretaceous eutherians. The premolariform ultimate lower premolar has a lager main cusp and small mesial and distal cuspules in *Cokotherium*, similar to that in *Juramaia*, *Acristatherium*, *Eomaia*, *Sasayamamylos* and Late Cretaceous asioryctitherians and cimolestids [6,8,9,11,49–51]. It is different from the morphology of the p5 in Late Cretaceous zhelestids, which is semimolariform with an incipient trigonid-talonid [48]. The Late Cretaceous zalambdalestids, have an even more derived ultimate lower premolar morphology with a two-cusped talonid [25,52]. The pattern of molarization of the premolars in *Cokotherium* probably represents the initial stage of molarization in eutherians.

### (d) Dental replacement

Mammals typically show a diphyodont replacement pattern with either sequential or alternating replacement of deciduous teeth [2,53]. Among Mesozoic eutherians the dental replacement pattern is known in *Juramaia*, *Kennalestes* and zhelestids [6,48,51,54]. In the upper dentition of *Cokotherium*, the presence of the deciduous and permanent P3 suggests that the DP3 is the last shedding upper deciduous premolar and that P3 is the last erupted permanent upper premolar, a similar case as in *Juramaia*. Similar to *Juramaia* the DP3 is also larger than the P3 in *Cokotherium*. Compared to other mammaliaforms, this dental replacement pattern is similar to those of trechnotherians (e.g. *Zhangheotherium*, *Juramaia*, *Kennalestes*) in having an alternating replacement (P2-P4-P3) rather than an anteroposterior replacement. In the lower dentition of *Cokotherium*, all teeth are erupted except the left lower canine, either a permanent or deciduous tooth. In the lower dentition of Late Cretaceous zhelestids, Archibald & Averianov [48] proposed seven stages of dental replacement with the lower canine reported to be fully erupted after m1 but before m2 or m3 (stage IV). In *Cokotherium*, all lower molars are fully erupted and even show some abrasion. Given that the right lower canine is fully erupted we interpret the delayed eruption of the left lower canine as pathological. The unerupted left lower canine is much smaller than that on the right side (with a length of 0.76 mm on the left, 1.04 mm on the right) which might indicate that it is a deciduous canine. In this scenario, the eruption and shedding of the deciduous left lower canine could have been interrupted and affected the eruption of the permanent tooth in this locus. It is also possible that the left canine is a permanent tooth and that the size difference between the lower canines could be explained by pathological tooth deformity. In humans, delayed eruption is most commonly seen in maxillary canines (upper canines) and distal upper molars (M3), followed by the mandibular canines (lower canines) [55,56]. Various factors can be involved in abnormal tooth development depending on local or systemic conditions, such as: physical obstruction, nutritional deficiency or a scar from trauma [57]. The defect in *Cokotherium* seems localized and thus, physical obstruction and nutritional deficiency might not be the major contributors to the abnormal tooth development. The delayed eruption might be best explained by an injury, although we cannot confirm any substantial injury to the mandible in this specimen.

### (e) Phylogeny

**Figure 4.**
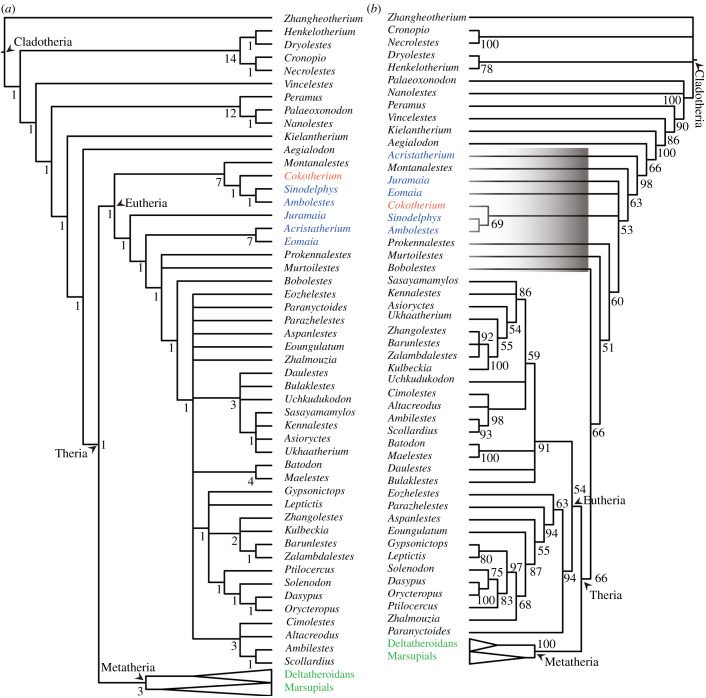
Simplified tree topologies of cladotherians based on (*a*) parsimony analysis (strict consensus tree of 15 most-parsimonious trees, tree length = 1810, consistency index = 0.313, retention index = 0.595) and (*b*) undated Bayesian analyses (50% majority-rule consensus). *Cokotherium* is marked in red, other eutherians discovered in Yanliao and Jehol Biota are marked in blue, and metatherians are marked in green. In the Bayesian undated tree (*b*), the black rectangle denotes taxa generally considered as basal eutherians that are here placed outside of Theria. In both analyses *Cokotherium* is most closely related to *Ambolestes* and *Sinodelphys*. Bremer support values and posterior probabilities are used for node support in (*a*) and (*b*), respectively.

Our parsimony analysis of 401 characters and 65 taxa returned a tree topology generally similar to that of Bi *et al*. [10]. *Cokotherium* is placed with *Montanalestes*, *Sinodelphys* and *Ambolestes* in a basal position within Eutheria, forming a sister clade to other eutherians (figure 4*a*) in which *Juramai*a, *Acristatherium* and *Eomaia* are revealed as most basal taxa. The monophyly of eutherians is not well supported with a low Bremer value compared to metatherians in the strict consensus tree. By contrast, our undated Bayesian analysis recovers a very different topology with several basal eutherians, including *Cokotherium*, placed outside of Theria (figure 4*b*). We find this topology less convincing and follow the results of the parsimony analysis here. Placement of Early Cretaceous and Jurassic taxa outside of Theria rather than within Eutheria would have broad implications for the timing of the divergence of metatherians and eutherians. The discrepancy between fossil and molecular data on the metatherian–eutherian divergence might not only be influenced by the still sparse fossil record of basal metatherians and eutherians but also the performance of different phylogenetic methods.

## 5. Conclusion

A new eutherian, *Cokotherium jiufotangensis*, is established based on a partial skeleton from the Jiufotang Formation. *Cokotherium* provides, to our knowledge, the first evidence of the ossified Meckelian cartilage in eutherians. The presence of a gracile ossified Meckelian cartilage in *Cokotherium* indicates an intermediate step between the Meckelian-attached middle ear of eutriconodontans and the detached middle ear of extant therians. It suggests that the underlying detachment between the ossified Meckelian cartilage and the middle ear took place concurrently with the gradual reduction of the ossified Meckelian cartilage in eutherians. Three-dimensional reconstruction of the inner ear morphology in *Cokotherium* reveals its mosaic morphology between stem therians and Late Cretaceous therians. *Cokotherium* has a molarized ultimate upper premolar (with three roots, a lingual protoconal swelling and a metaconal swelling) and a non-molarized ultimate lower premolar, probably representing an initial stage of molarization in eutherian premolars. The parsimony analyses places *Cokotherium* as a member of the most basal clade within Eutheria. This and other discoveries of early therians are essential for reconstructing the early evolutionary history of therians but are still insufficient for elucidating the discrepancy between metatherian–eutherian divergence times based on fossil and molecular data.

## Data accessibility

The datasets supporting this article have been uploaded as the electronic supplementary material [58].
